# Lagrangian for Circuits with Higher-Order Elements

**DOI:** 10.3390/e21111059

**Published:** 2019-10-29

**Authors:** Zdenek Biolek, Dalibor Biolek, Viera Biolkova

**Affiliations:** 1 Department of Microelectronics, Brno University of Technology, 616 00 Brno, Czech Republic; zdenek.biolek@gmail.com (Z.B.); dalibor.biolek@unob.cz (D.B.); 2 Department of Electrical Engineering, University of Defence, 662 10 Brno, Czech Republic; 3 Department of Radio Electronics, Brno University of Technology, 616 00 Brno, Czech Republic

**Keywords:** Hamilton’s variational principle, higher-order element, memristor, Lagrangian, Chua’s table, Euler–Lagrange equation

## Abstract

The necessary and sufficient conditions of the validity of Hamilton’s variational principle for circuits consisting of (*α*,*β*) elements from Chua’s periodical table are derived. It is shown that the principle holds if and only if all the circuit elements lie on the so-called Σ-diagonal with a constant sum of the indices *α* and *β*. In this case, the Lagrangian is the sum of the state functions of the elements of the *L* or ^+^*R* types minus the sum of the state functions of the elements of the *C* or ^−^*R* types. The equations of motion generated by this Lagrangian are always of even-order. If all the elements are linear, the equations of motion contain only even-order derivatives of the independent variable. Conclusions are illustrated on an example of the synthesis of the Pais–Uhlenbeck oscillator via the elements from Chua’s table.

## 1. Introduction

It is well known that the Lagrange formalism adopted from classical mechanics [1] can be used for describing phenomena in electric circuits. The equations of motion of a conservative system can be derived from the Lagrange function

(1) $$L=L\left(x_{1},..,x_{n},{\dot{x}}_{1},..,{\dot{x}}_{n},t\right)$$

according to the Euler–Lagrange equation

(2) $$\frac{d}{dt}\left(\frac{\partial L}{\partial {\dot{x}}_{i}}\right)-\frac{\partial L}{\partial x_{i}}=0$$

Here, *x_i_*, for *i* = 1,..., *n*, are the generalized coordinates of the system. The dot above *x_i_* denotes the derivative with respect to time, or, in other words, the corresponding component of the generalized velocity, and *n*∈ Z+ is the number of degrees of freedom. The Lagrange function is usually derived as a difference between the kinetic and the potential energy of the system, or the energy of inductors and capacitors in electrical engineering. In the latter case, the electric charges or integrals of the electric voltages, the fluxes, usually supersede the role of the generalized coordinates. The coordinates are selected depending on the method of the circuit analysis. The generalized velocities are the derivatives of the coordinates with respect to time, thus electric currents and voltages. Systematic differentiation of the Lagrange function (1) with respect to coordinates, velocities, and time, yields the equations of motion (2) expressing the equilibrium of the generalized forces. For electrical engineering, these equations represent Kirchhoff’s voltage or current laws (the KVL or KCL).

The so-called Hamilton variational principle holds for the conservative systems. Let the system go through the states *x_i_*(*t*_1_) and *x_i_*(*t*_2_), *i* = 1,..., *n,* at instants *t*_1_ and *t*_2_, *t*_2_ > *t*_1_. The principle states that, within the time interval (*t*_1_,*t*_2_), the system “selects” for its motion, between the initial and the final state, such a trajectory that is a stationary point of the action functional

(3) $$A=\int_{t_{1}}^{t_{2}}L\left(x_{1},..,x_{n},{\dot{x}}_{1},..,{\dot{x}}_{n},t\right)dt$$

The functional (3) is a definite integral, which generates a number for each trajectory. From all the conceivable trajectories that might pass through the terminal states at instants *t*_1_ and *t*_2_, it is only for the existing trajectory that the action (3) takes the stationary value (the minimum, maximum, or saddle point). That is why Hamilton’s principle is denoted as the principle of stationary action. Any virtual variation (*δx*_1_,..., *δx_n_*) of the existing trajectory must give rise to a zero variation of the first order *δA* of the action. The existing trajectory is, therefore, the solution of the optimization task

(4) $$\delta A=\delta \int_{t_{1}}^{t_{2}}Ldt=0$$

It can be easily proven that the conditions (2) and (4) are equivalent [1]. If the system fulfils the Hamilton’s variational principle (4), then its equations of motion are in the form of the Euler–Lagrange Equation (2) and vice versa.

External excitation can be included in the modified Lagrange function *L’* as

(5) $$L^{\prime }=L+Q_{1}x_{1}+..+Q_{n}x_{n}$$

where *Q*_1_,..., *Q_n_* are components of the generalized external force. Then Hamilton’s variational principle also holds for systems represented by this modified Lagrange function.

The presence of arbitrary dissipative forces in the system, dependent on generalized velocities, means a violation of the necessary condition for the validity of Hamilton’s variational principle. Additional terms, incompatible with the desired form (2), appear in the equations of motion. The dissipative forces are usually derived as gradients of the dissipative function *D*, which depends on generalized velocities. The equations of motion are then in the form

(6) $$\frac{d}{dt}\left(\frac{\partial L}{\partial {\dot{x}}_{i}}\right)-\frac{\partial L}{\partial x_{i}}+\frac{\partial D}{\partial {\dot{x}}_{i}}=0$$

It is evident from (6) that not all the generalized forces can be generated from a single scalar function *L* as Hamilton’s principle states. The dissipative forces must be generated via another scalar function, the dissipative function *D*.

The utilization of classical Lagrange formalism leads to equations of motion (2), which are of the 2^nd^ order. It is a logical consequence of the fact that the equations of motion of classical mechanics represent an equilibrium of inertial, potential, and dissipative forces. However, some existing basic higher-order processes are not governed by this simple rule. The interest in studying systems whose dynamics explicitly depends on the accelerations and higher-order derivatives of generalized coordinates with respect to time gave rise to Ostrogradsky’s work [2] (1850), which generalizes the Lagrange and Hamilton formalism in this sense. Other works such as [3] by Whittaker (1904) followed [2], introducing the Lagrange function in the form

(7) $$L=L\left(x_{1},..,x_{n},x_{1}^{\left(1\right)},..,x_{1}^{\left(1\right)},..,x_{1}^{\left(m\right)},..,x_{1}^{\left(m\right)},t\right)$$

where *m* is the maximum order of the derivative of a generalized coordinate with respect to time. The equations of motion are as follows:

(8) $$\frac{\partial L}{\partial x_{i}}-\frac{d}{dt}\left(\frac{\partial L}{\partial {\dot{x}}_{i}}\right)+\frac{d^{2}}{dt^{2}}\left(\frac{\partial L}{\partial x_{i}^{2}}\right)-..+{\left(-1\right)}^{m}\frac{d^{m}}{dt^{m}}\left(\frac{\partial L}{\partial x_{i}^{m}}\right)=0$$

The interest in higher-order Lagrangians of type (7) is currently growing due to the dynamics of higher-order systems in various branches of theoretical physics, control theory, and applied mathematics being intensively studied. A brief survey is given in [4].

A similar development has also been noted in the theory of electrical circuits, stigmatized to some extent by the idea of the exclusivity of three fundamental elements, namely the inductor, capacitor, and resistor. A plethora of novel two-terminal devices with startling parameters and intriguing application potentials, such as the ovonic threshold switch, Josephson junction, or various diodes (Esaki, Gunn, IMPact ionization Avalanche Transit-Time (IMPATT)), appeared in the 1960s. Researchers in the field of nonlinear electronics were then faced with phenomena that were difficult to categorize within the current circuit theory, or they were classified as “anomalies”, “exotic”, or “paradox” [5]. It was a period when the intellectual basis of a new scheme began to be formed that would put all the above phenomena in order.

The axiomatic basis of the nonlinear circuit theory was laid down in 1969 by Leon Chua in the book [6]. The principles in [6] contributed to the prediction of a memristor as the fourth fundamental electrical element in 1971 [7]. In the concept of the so-called periodical table of fundamental elements from 1980 [8], the resistor, capacitor, inductor, and memristor are just special cases of more general (*α*,*β*) elements, also denoted as the HOEs (Higher-Order Elements). They preserve an unambiguous relationship between the variables *v*^(^*^α^*^)^ and *i*^(*β*)^, where the indices *α*,*β* are the orders of derivatives/integrals (for positive/negative indices) of the terminal voltages *v* and currents *i* with respect to time. Until then, elements occupied the following positions in the new schematics: (0,0)—resistor (R), (0,−1)—capacitor (C), (−1,0)—inductor (L), and (−1,−1)—memristor (MR). After discovering the nanodevice with a memristive behavior in the Hewlett–Packard laboratories in 2008 [9], Chua appealed to search for the other missing elements from the table [10], namely the memcapacitor (MC) (−1,−2) and the meminductor (ML) (−2,−1). The electrical elements from the family of the so-called Frequency-Dependent Negative Resistors (FDNRs), known since the 1960s and gradually synthetized as active elements with the help of Bruton’s work [11], behave as elements from Chua’s table. The work [12] distinguishes between the FDNC (Frequency-Dependent Negative Conductor) and the FDNR (Frequency-Dependent Negative Resistor). Such resistors with negative resistances, providing quadratic dependence of the small-signal conductance or resistance on the frequency, are the (1,−1) or (−1,1) elements from Chua’s table. In [13], Soliman introduces the FDPC (Frequency-Dependent Positive Conductance), whose conductance depends on the fourth power of frequency. In fact, it is the (2,−2) element. The mechanical inerter from 2002 [14] was recognized as the (1,0) element [15].

Some recent papers suggest modifications of the original Chua’s table of fundamental elements [16,17,18]. The so-called storeyed structure of the HOEs [16] just transforms Chua’s table into a different graphic form [16]. The storeyed structure and Chua’s table, therefore, contain identical sets of the HOEs. Wang’s triangular periodic table [17] contains only HOEs that occupy only three so-called Δ-diagonals of the original Chua’s table (see Section 2) for Δ = 1, 0, and 1. Since no other HOEs, the negative resistors being among them, are missing in Wang’s table, this table cannot be utilized for our purposes. It is well documented via the impossibility of drawing a Lagrangian for circuits with dissipative elements [19].

The reason why Chua’s and Wang’s table contain different sets of elements consists in different conceptions of the fundamental element. While Chua’s definition starts from an unambiguous axiomatic approach [8], Wang introduces additional limiting conditions, which must be fulfilled in order to denote the element as fundamental. These limitations are of postulating nature, namely: The fundamental electric quantities are the flux and the charge. A fundamental circuit element should link two electric quantities, at least one of which should be fundamental [17]. Such a tapered definition of the fundamental element results in the absence of quite a few of the HOEs in Wang’s table. However, these missing elements are unsubstitutable in modeling the existing dynamic systems [20].

The work [18], starting from the intuitive approach, re-defines the fundamental element even more. For example, according to [18], the fundamental elements must be linear. Such a premise must inevitably exclude the memristor, whose constitutive relation is nonlinear, from the set of fundamental elements. Since the corresponding table of elements suggested in [18] contains only a subset of linear elements, it cannot be used for studying the variational principles in dynamic systems containing the HOEs.

For the above reasons, this work will, therefore, be based on Chua’s approach.

The Chua’s table of fundamental elements became an important tool of the so-called predictive modeling [20]. In spite of some limits of this approach [21], it can be advantageous in the analysis and also synthesis, provided that the system be built exclusively from HOEs as the basic building blocks. One limit is due to the fact that only two-terminal HOEs appear in Chua’s table. In order to model a certain class of systems, the above idea of two-terminal fundamental elements should, therefore, be generalized to multipoles or multiports [22]. The importance of predictive modeling is increasing when studying systems with higher-order dynamics. It can be documented in researching chaotic phenomena, where the higher-order dynamics is a necessary condition. According to [23], 427 important papers about the analysis or synthesis of chaotic circuits via memristors, memcapacitors, and meminductors were published within one decade after discovering the memristor in Hewlett-Packard labs in 2008 [9], 283 from them within the last three years of that decade.

In spite of such a precipitate development, two basic questions have still not been answered: Can the Lagrangian (7) of a system compounded from HOEs be constructed and under what conditions, and does Hamilton’s variational principle hold for such systems. The first responses to these questions were the studies [24,25,26], which introduced Lagrange and Hamilton formalisms for circuits with memristors and memcapacitors. The follow-up paper [27] defines the state functions of general HOEs and derives the Euler–Lagrange equations from them. It also states in [27] that “The paper does not deal with the question of whether or not the extremal principle connected with the original idea of the Lagrangian is fulfilled.” This paper aims to answer the above questions. Section 2 summarizes the current classification of the fundamental elements according to their positions on the Δ-diagonals of the table and the definitions of their state functions according to [27]. Recalled therein are some rules of the taxicab geometry, which hold for the table of elements, recently published in [28]. The following key Section 3 describes the derivation of Hamilton’s variational principle for circuits containing elements from an arbitrary Σ-diagonal of the table. Section 4 illustrates that the principle does not hold for circuits containing elements from various Σ-diagonals. Section 5 transforms the Lagrangian of the circuit with HOEs into a form that is compatible with the hitherto used forms of the classical potential functions. In Section 6, the results are applied to the analysis and synthesis of the Pais–Uhlenbeck oscillator via HOEs.

## 2. Table of Higher-Order Elements

**Figure 1:**
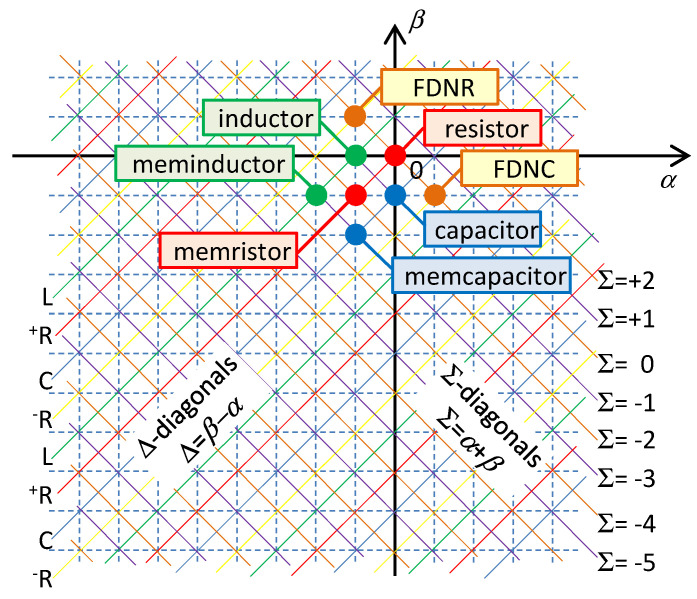
Most currently known fundamental elements from Chua’s table. Σ-diagonals provide the constant sum of indices *α* + *β*, Δ-diagonals the constant difference *β* – *α*. Σ-diagonals are occupied by resistors (even Σ, orange color) or reactive elements (odd Σ, violate color), the ^+^*R*, *L*, ^−^*R*, and *C* elements periodically alternate on Δ-diagonals (red, green, orange, and blue colors).

The basic attributes of Chua’s table of HOEs are well known from the original paper [8]. The elements are drawn in Figure 1 as circles with the integer-type (*α*,*β*) coordinates in the rectangular grid.

Every element is one-port, which predicts an unambiguous relation between its port variables *v*^(*α*)^ and *i*^(*β*)^, i.e., it preserves the constitutive relation *F*(*v*^(*α*)^,*i*^(*β*)^) = 0, where *F*() is generally a nonlinear function, in all circumstances and regardless of the behavior of the surrounding network. The elements with explicit forms of the constitutive relation.

(9) $$v^{\left(\alpha \right)}=f\left(i^{\left(\beta \right)}\right),\mathrm{or}i^{\left(\beta \right)}=g\left(v^{\left(\alpha \right)}\right)$$

are called current- or voltage-controlled elements.

The (*α*,*β*) coordinates are not the only way of defining the location of the element in the table. There may be other coordinates (Σ,Δ), where Σ = *α* + *β* and Δ = *β* − *α*. All elements with the same value Σ/Δ lie on one of the Σ/Δ-diagonals; see Figure 1. The (Σ,Δ) element is located at the intersection of both diagonals.

It is well known that the Δ-diagonals determine the small-signal behavior of the elements located on these diagonals [8]. Consider the voltage representation of the element according to (9), and analyze the small-signal deflections of voltages and currents *dv* and *di* around the operating point *Q* lying on the constitutive relation. It is obvious that

(10) $$dv^{\left(\alpha \right)}=m_{Q}di^{\left(\beta \right)}$$

where *m_Q_* is the slope of the constitutive relation at point *Q*. The Fourier transform of Equation (10) and a simple rearrangement yield the formula for the small-signal impedance of the element

(11) $$Z\left(j\omega \right)={\left(j\omega \right)}^{\Delta }m_{Q}$$

It follows from (11) that elements lying on a given Δ-diagonal have the same small-signal characters. For Δ repeatedly increasing by one, the element type will change with a period of 4 among the positive resistor ^+^*R*, inductor *L*, negative resistor ^−^*R*, and the capacitor *C*. For all these cases, the elements are generally frequency-dependent according to (11).

**Figure 2:**
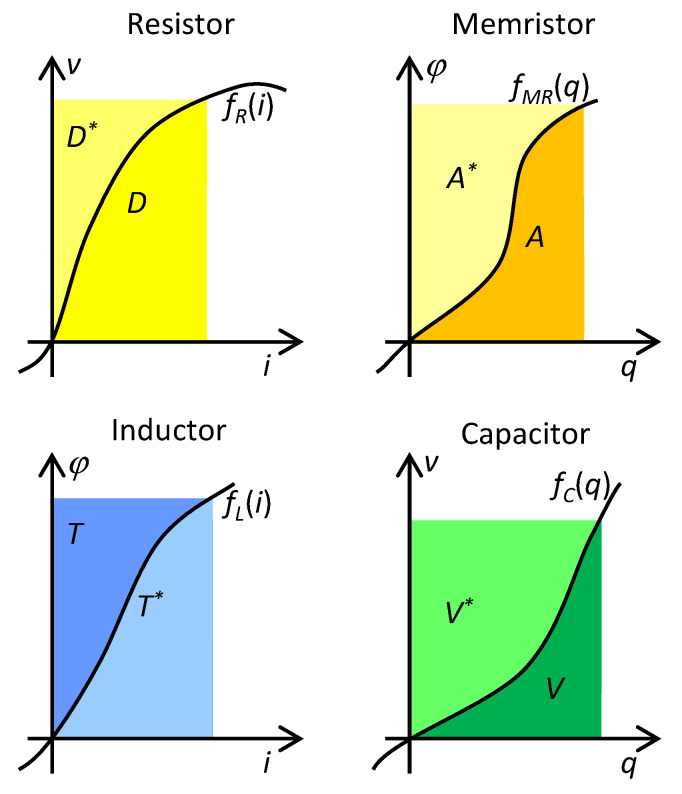
State functions and cofunctions of four fundamental elements. D = dissipative function (content), A = action, T = energy of the magnetic field of the inductor, V = energy of the electrostatic field of capacitor. The cofunctions are denoted by asterisks.

Considering small-signal modeling, it follows from Figure 1 that a given Σ-diagonal can be occupied either by resistors (regularly alternating positive and negative resistors for even values of Σ), or by reactive elements (regularly alternating inductors and capacitors for odd values of Σ). It is also known that the number Σ determines the physical dimension of the corresponding state function [27]. The dissipative function with the dimension of power is a state function of resistors from the diagonal Σ = 0. The state functions of the inductors and capacitors from the diagonal Σ = −1 are energies, and the state function of the memristor (Σ = −2) is called action [7], i.e., the time integral of the energy. All these functions are defined in the same manner, thus as integrals of the constitutive relation of the element with respect to the corresponding constitutive variable. It means that they can be illustrated via the areas below the graphs of given constitutive relations; see Figure 2. The pair of state functions originating by the integration with respect to one or the other constitutive variable have been denoted in physics and electrical engineering as a function and co-function. In 1951, Cherry and Miller introduced the notions of energy and coenergy [29] for the reactive elements and the power functions content and cocontent [30] for resistive elements. The functions and co-functions are assigned to individual representations of the state functions of the four fundamental electrical elements, according to Table 1 [31].

The generalization of state functions and cofunctions to arbitrary (*α*,*β*) elements is introduced in [27]. The state functions are integrals of the corresponding constitutive relations

(12) $$S_{\alpha ,\beta }=\int v^{\left(\alpha \right)}di^{\left(\beta \right)}\mathrm{or}{\hat{S}}_{\alpha ,\beta }=\int i^{\left(\beta \right)}dv^{\left(\alpha \right)}$$

for the current or voltage representation of the element. The mechanism for classifying the representation (12) into functions and cofunctions is obvious from Table 1.

It follows from the definition formulae (12) that the physical dimension of the state function of a general (*α*,*β*) element is [Volt⋅Amper⋅sec^−Σ^] or [Joule⋅sec^–(Σ+1)^]. The state functions of all the elements located on the common Σ-diagonal have, therefore, the same physical dimension. It is a necessary condition for their additivity.

Let the symbols T^*^ and *V* or ^+^*D* and ^–^D^*^ denote the sums of state functions T^*^_α__,*β*_ and *V_α_*_,*β*_ of type *L* and type *C* element, or the functions ^+^*D_α_*_,*β*_ and ^−^D^*^_α__,*β*_ of type ^+^*R* and ^–^*R* elements from one Σ-diagonal, i.e.,

(13a) $$T^{*}=\sum T_{\alpha ,\beta }^{*},V=\sum V_{\alpha ,\beta }$$

or

(13b) D+=∑Dα,β+,D*−=∑Dα,β*−.

The summations are done for all circuit elements of *L* and *C* or ^+^*R* and ^–^*R* types from a given Σ-diagonal. Seeing that we work with generalized coordinates, which can be of various physical dimensions, the quantities (13a) and (13b) can be regarded as generalized energies and contents, respectively.

**Figure 3:**
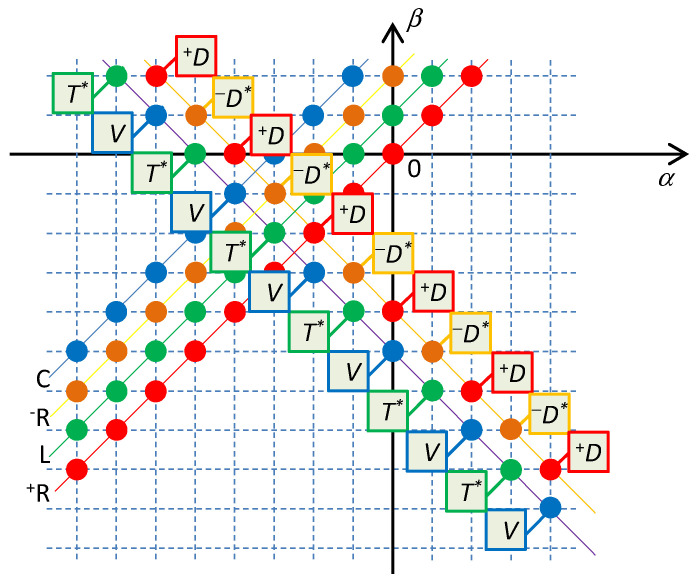
Potential functions and cofunctions for the current representation of elements. Dissipative functions ^+^*D_α_*_,*β*_ and cofunctions ^−^D^*^_α__,*β*_ alternate on Σ-diagonals with resistive elements. Potential functions of capacitors *V_α_*_,*β*_ and cofunctions of inductors T^*^_α__,*β*_ alternate on Σ-diagonals with reactive elements. For lucidity, the subscripts of the potential functions are omitted.

The regular alternation of potential functions and cofunctions within the even (^+^*D* and ^–^D^*^ for elements of ^+^*R* and ^–^*R* types) and odd (T^*^ and *V* for elements of *L* and *C* types) Σ-diagonals is demonstrated in Figure 3.

For the current representation of state function (12), the voltage variable *v*^(*α*)^ = *dS_α_*_,*β*_/*di*^(*β*)^ is equal to the slope of the state function of the element at the operating point. Considering only the elements within one given Σ-diagonal, each of them will have a unique index *β*. The voltage quantity *v*^(*α*)^ of a specific element will be equal to the partial derivative of the corresponding *total* state functions (13a) or (13b) of the circuit with respect to a specific current variable *i*^(*β*)^.

**Figure 4:**
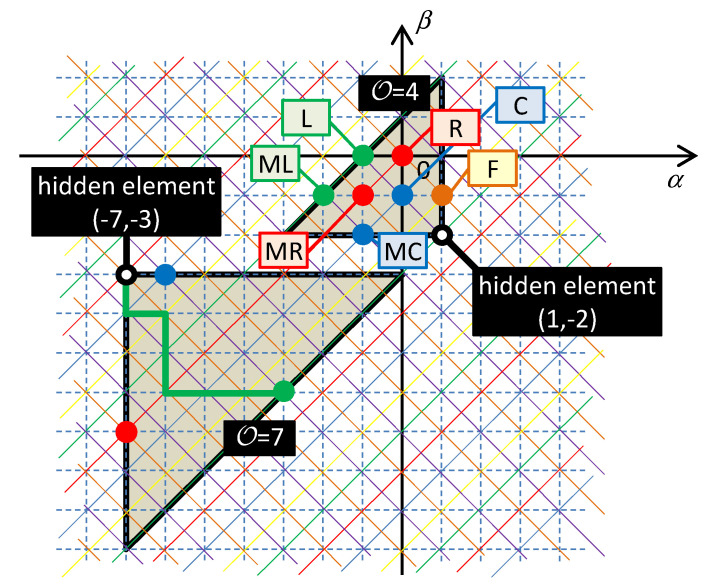
Characteristic quarter-circles for the series connection of *R*-*L*-*C*-*MR*-*ML*-*MC*-*FDNC* elements (up) and for the parallel connection of three different elements (down). The taxicab quarter-circle with the center in the hidden element has the form of one of four isosceles triangles, which originate by partitioning the rectangular, representing the taxicab circle, by its diagonals. The order 𝒪 is given by the element, which is the farthest from the hidden element and is equal to this distance (the green path corresponding to 𝒪 = 7).

The rules of the Taxicab geometry hold in the table of fundamental elements. According to these rules, the distance between the elements is given by the shortest path between them in the frame of the rectangular net. In such a type of geometry, the circle drawn around a central point has the form of a square whose diagonals occupy the horizontal and vertical positions. According to [28], the order 𝒪 of the differential equation describing the behavior of one-port consisting of serial and parallel combinations of HOEs is equal to the radius of the smallest quarter-circle that incepts all the elements of the one-port. The center of the quarter-circle is occupied by the so-called hidden element with the coordinates (*α*_MAX_,*β*_MIN_) or (*α*_MIN_,*β*_MAX_) for a series or a parallel connection of the elements. The subscripts MAX and MIN denote the maximum and minimum coordinates that are occupied by the elements in the table. An example of constructing the quarter-circles for series and parallel connections of HOEs is shown in Figure 4.

The equation of motion of the series connection of HOEs expresses the KVL for the generalized voltage *v*^(*α*MAX)^ or the KV^(*α*MAX)^L in the form

(14) $$\sum_{j=0}^{m}f_{j}^{\left(\alpha_{\mathrm{MAX}}-\alpha_{j}\right)}\left(i^{\left(\beta_{j}\right)}\right)=v^{\left(\alpha_{\mathrm{MAX}}\right)}$$

where the index *j* = 0 corresponds to the hidden element, *v* is the terminal voltage, *m* is the number of elements increased by the hidden element if appropriate, *f_i_*() is the constitutive relation (9) of the *i*^th^ element, *i* = 0, ..., *m*. Note that, according to Figure 4, the order *β_j_* of the derivative of current in differential equation (14) will take values from *β*_MIN_ to (*β*_MIN_ + 𝒪). Thus, it is natural to introduce a new variable *x* = *i*^(*β*MIN)^, and the differential equation drawn for such a generalized current will contain its derivatives of orders 0 to 𝒪, which will correspond to the distances of the elements from the hidden element in vertical direction. Similarly, let us introduce the generalized voltage *u* = *v*^(*α*MAX)^. According to (14), the orders of derivatives of generalized voltages *f_j_* will also be between 0 and 𝒪 since they will correspond to the distances of the elements from the hidden element in the horizontal direction.

The symbols *x* and *u* can also be universally used for generalized voltages and currents of elements connected in parallel. The situation for a series and parallel connection is summarized in Table 2. For a parallel connection, where KC^(*β*)^L is applied, the driving variable is *u* = *i*^(*β*MAX)^ flowing in the common node. The individual terms of differential equations will signify the generalized currents *u_j_* = *i_j_*^(*β*MAX)^ flowing through individual elements.

The uniform notation enables choosing both generalized voltages and currents as the generalized coordinates *x_i_* in the Lagrangian (7) and in the corresponding equations of motion (8).

The circuits with HOEs, where Hamilton’s principle holds are governed by the set of differential equations (8). Consider that each equation represents KV^(*α*)^L or KC^(*β*)^L for some loop or node. Then the variables *x_i_*, *i* = 1,..., *n*, will be loop variables of *i*^(*β*)^ type or nodal variables of *v*^(*α*)^ type, respectively.

The Lagrange formalism (7) and (8) is based on the generalized coordinates *x_i_*. On the other hand, predictive modeling starts from different variables, namely from generalized voltages and currents of individual elements joined by the constitutive relations (9). In order to apply the Lagrange formalism to circuits with HOEs, the transform between these two sets of variables must be used. The following procedure is a generalization of the method described in [19]. Consider the above transform as a linear combination of the variables *x_i_*

(15) xε=∑i=1naiεxi

where *x_ε_* is the generalized voltage or current of a specific element *ε*, *^ε^a_i_* are the coefficients of the linear combination for the element *ε*. For example, if the element coincides with the loops No. 1 and 3, the corresponding coefficients *^ε^a*_1_ and *^ε^a*_3_ will be +1 or −1 according to the agreement or disagreement of the reference directions of the element and of the loop. If the element does not coincide with the loop, the corresponding coefficient is zero. A similar consideration applies to the nodal variables—the element *ε* directed between nodes 2 and 4 provides the coefficients *^ε^a*_2_ = +1 and *^ε^a*_4_ = −1, and all the other coefficients are zero.

The symbol *ε* will be specified as follows: all the elements from the circuit will be classified according to their positions in Chua’s table (more elements of the same type will be counted as one element) and numbered from 0 to *m*. Zero will be assigned to the hidden element. Each element from the circuit will belong to one type from the set *ε_k_*, *k* = 0, ..., *m*.

## 3. Sufficient Condition for Hamilton’s Principle in Circuits with HOEs

In this Section, the following proposition will be proved:

**Proposition** **1.** *If the circuit is composed exclusively of the elements from Chua’s table that are located on the common Σ-diagonal, then it conforms to Hamilton’s principle.*

The proof will be given for the KV^(*α*)^L representation of the circuit, according to Table 2. The proof for the dual KC^(*β*)^L representation is analogous.

**Figure 5:**
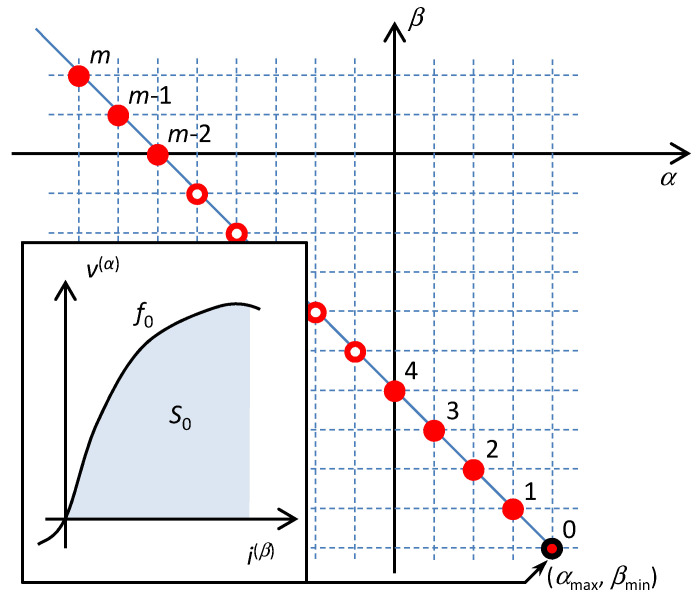
The loop in the circuit is formed by elements appearing on the common Σ-diagonal. The elements, numbered from 0 to *m*, are characterized by constitutive relations *f*_0_ to *f_m_*. The corresponding state functions *S*_0_ to *S_m_* signify the areas below the constitutive relations.

Consider a loop in the circuit containing the (*α*,*β*) elements that are located on a general Σ-diagonal in Figure 5. The elements are numbered in ascending order from 0 to *m*, starting with the hidden element (*α*_MAX_,*β*_MIN_).

The constitutive relations and the state functions of the elements of *ε_k_* type, *k* = 0,1,2,…, *m*, are

(16) $$v^{\left(\alpha_{k}\right)}=f_{k}\left(i^{\left(\beta_{k}\right)}\right),S_{k}\left(i^{\left(\beta_{k}\right)}\right)=\int f_{k}\left(i^{\left(\beta_{k}\right)}\right)di^{\left(\beta_{k}\right)}$$

If the notation for generalized voltages and currents for KV^(*α*)^L from Table 2 is taken into consideration, then

(17) $$S_{k}\left(i^{\left(\beta_{k}\right)}\right)=S_{k}\left(x^{\left(\beta_{k}-\beta_{\mathrm{MIN}}\right)}\right)=S_{k}\left(x^{\left(k\right)}\right)$$

Equation (17) follows from the fact that the distance of the *k*^th^ element from the hidden element along the *β* coordinate is equal to the number *k* (Considering the position of the element on the diagonal, the same holds for the *α* coordinate.) The formal variable *x* in (17) can be substituted by the generalized current of a specific element *ε*, which is of *ε_k_* type with the constitutive relation *f_k_*().

Let us analyze the impact of the trajectory variation *δx_i_*, *i* = 1,..., *n*, on the state functions *S_k_*. It follows from the definition (16) of the state function that

(18) $$\delta S_{k}=f_{k}\left(x^{\left(k\right)}\right)\delta x^{\left(k\right)}$$

For a particular element *ε*, the variation *δx*^(*k*)^ will depend on which loops the element will be involved in. Considering Equation (15), it holds

(19) δSk=∑i=1nfkiδxi(k),fki=aiεfk

The index *i* of the term *^i^f_k_* indicates that the constitutive relation is completed by a correct sign according to the reference direction of the element within the *i*^th^ loop, or it makes this relation zero if the element is not present in the loop.

The variations of the coordinates, not their derivatives, appear in the variational task (4). In the next step, the variations *δx_i_*^(*k*)^ will, therefore, be transferred to *δx_i_*. This well-known procedure via repetitive integration of (19) by parts yields the result

(20) δ∫t1t2Skdt=∑i=1n[∑j=0k−1(−1)k−1−jfk(k−1−j)iδxi(j)]t1t2+(−1)k∑i=1n∫t1t2fk(k)iδxidt

If the boundary points of the original trajectory and the first derivatives of the (*k*−1)^th^ orders are preserved, it follows from (15) that the first right-side term of (20) must be zero. Summing the terms in (20) separately for odd and for even indices *k* and the calculating their difference will result in

(21) δ∫t1t2(∑k=evenSk−∑k=oddSk)dt=∫t1t2∑i=1n(∑k=0mfk(k)i)δxidt

An arbitrary number of elements with the constitutive functions *f_k_* and state functions *S_k_* can be present in the circuit. The summation of Equation (21) for all the elements *ε* in the circuits leads to the formula

(22) δ∫t1t2∑ε(∑k=evenSk−∑k=oddSk)dt=∫t1t2∑i=1n(∑k=0m(∑εfk(k)i(xε(k))))δxidt

The sum with the summation index *k* in (22) represents KV^(*α*)^L for the *i*^th^ loop, or one of the Euler–Lagrange equations (8). The formula (22) is therefore equal to zero, and the left-side integrand is the Lagrange function of the entire circuit. Then, if the boundary conditions

(23) $$\delta x_{i}\left(t_{1}\right)=\delta x_{i}\left(t_{2}\right)=0,..,\delta x_{i}^{\left(m-1\right)}\left(t_{1}\right)=\delta x_{i}^{\left(m-1\right)}\left(t_{2}\right)=0$$

are fulfilled for every *i* = 1,…, *n*, then the existing trajectory is an extremal of the action potential, or

(24) $$\delta A=\delta \int_{t_{1}}^{t_{2}}L\left(x_{1},x_{1}^{\left(1\right)},..,x_{1}^{\left(m\right)},..,x_{n},x_{n}^{\left(1\right)},..,x_{n}^{\left(m\right)}\right)=0$$

where

(25) $$L=\sum_{\varepsilon }\left(S_{0}-S_{1}+S_{2}-S_{3}+..+{\left(-1\right)}^{m}S_{m}\right)$$

The following differential equation holds for a series circuit with one loop in which just one element of each type occurs:

(26) $$f_{0}\left(x\right)+f_{1}^{\left(1\right)}\left(x^{\left(1\right)}\right)+..+f_{m}^{\left(m\right)}\left(x^{\left(m\right)}\right)=0$$

Note that only the even-order derivatives of the independent variable are present in the differential equation of the linear circuit in which Hamilton’s principle applies. The reason is that if the constitutive relations *f_k_* from (22) are linear, then it holds

(27) $$f_{k}^{\left(k\right)}\left(x_{\varepsilon }^{\left(k\right)}\right)=m_{k}x_{\varepsilon }^{\left(2k\right)}$$

where *m_k_* is the slope of the constitutive relation at the operating point.

**Figure 6:**
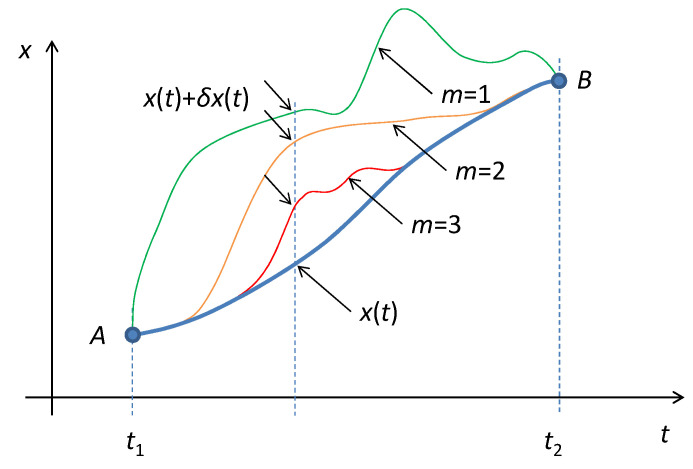
Illustration of the variations of an actual trajectory for various values of *m*. The virtual trajectories *x*(*t*) + *δx*(*t*) retain the curvature of the original trajectory *x*(*t*) up to the (*m* − 1)^th^ order at the boundary points.

The boundary conditions (23) indicate that the trajectory variation is ruled by stricter conditions than for the classical variational principle. All virtual trajectories must copy the first (*m*−1) orders of the curvatures of the original trajectory at both boundary points. Therefore, the variational principle of systems containing HOEs retains fewer degrees of freedom of the variation of the trajectory, as indicated in Figure 6.

## 4. Necessary Condition for Hamilton’s Principle in Circuits with HOEs

Let us prove the following proposition:

**Proposition** **2.** *If a circuit, made up exclusively from the elements from Chua’s table, has to comply with Hamilton’s principle, then all its elements must occur on a common Σ-diagonal.*

**Figure 7:**
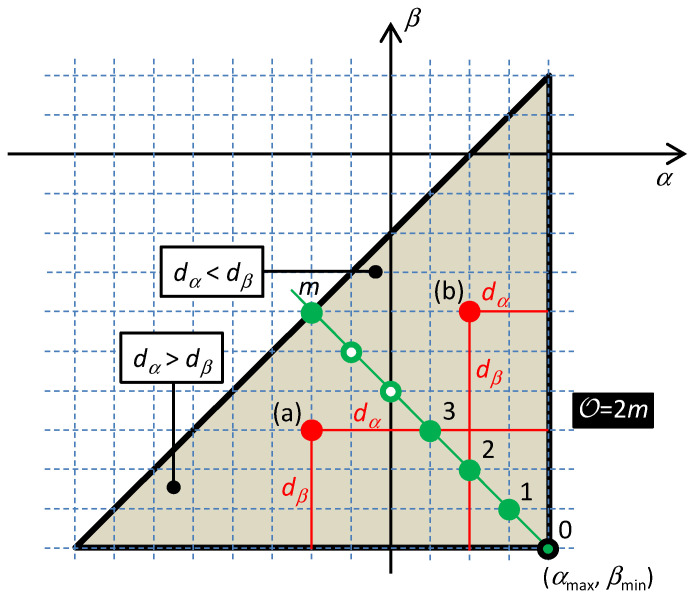
The loop is formed via interconnecting *m* + 1 elements located on a common Σ-diagonal (green circles) and one element (red circle) (**a**) below or (**b**) above the diagonal. The elements numbered from 0 to *m* are characterized by constitutive relations *f*_0_ to *f_m_*. The added element beyond the diagonal has the constitutive relation *f_add_*. The corresponding state functions are *S*_0_ to *S_m_* and *S_add_*.

Consider the contrary case: one element, *ε_add_*, with the constitutive relation *f_add_*() and state function *S_add_*, is located beyond the diagonal, as shown in Figure 7, case (a) or (b). Let its distance from the hidden element be *d_α_* + *d_β_*, where the subscripts denote the components of this distance along the *α* and the *β* coordinates. The distance of the element from the Σ-diagonal is

(28) $$d=\left|d_{\alpha }-d_{\beta }\right|$$

with the difference *d_α_* − *d_β_* being positive/negative below/above the diagonal, respectively. The constitutive relation *f_add_*() introduces the coupling condition

(29) $$u_{add}=f_{add}^{\left(d_{\alpha }\right)}\left(x_{add}^{\left(d_{\beta }\right)}\right)$$

KV^(*α*)^L for the *i*^th^ loop will be in the form

(30) fadd(dα)i(xadd(dβ))+∑k=0m(∑εfk(k)i(xε(k)))=0

If a Lagrangian existed that would generate the complete equations (30), then the following condition would have to hold:

(31) ∫t1t2∑i=1n(fadd(dα)i(xadd(dβ)))δxidt+∫t1t2∑i=1n(∑k=0m(∑εfk(k)i(xε(k))))δxidt=0

The first integral in (31) can be expanded via successive integrations by parts into the form

(32) ∫t1t2∑i=1nfadd(dα)iδxidt=∑i=1n[∑j=0dβ−1(−1)dβ−1−jfadd(dα−1−j)iδxi(j)]t1t2+(−1)dα∑i=1n∫t1t2fadd(dα−dβ)iδxidt

It follows from the range of the *d_α_* parameter that the first right-side sum is equal to zero if the boundary conditions (23) are fulfilled. The second sum can be written as follows:

(33) ∑i=1n∫t1t2fadd(dα−dβ)iδxidt=∫t1t2fadd(dα−dβ)δxadd(dβ)dt=∫t1t2δSadd(dα−dβ)dt

Seeing that *d* = |*d_α_* − *d_β_*| according to (28), Equation (33) contains a *d*-multiple integral of the state function *S_add_* of the element *ε_add_* with respect to time, or its time derivative, depending on whether the element appears above or below the Σ-diagonal at the distance *d* from it:

(34) $$S_{add}^{\left(d_{\alpha }-d_{\beta }\right)}=\{\begin{array}{ll} \frac{d^{d}S_{add}}{dt^{d}} & for\;d_{\alpha }>d_{\beta }\\ S_{add} & for\;d_{\alpha }=d_{\beta }\\ \underset{d}{\underset{︸}{\int ..\int }}S_{add}dt_{1}..dt_{n} & for\;d_{\alpha }<d_{\beta } \end{array}$$

The variation of the action is then

(35) $$\delta A=\int_{t_{1}}^{t_{2}}\left(\delta \left(S_{0}-S_{1}+..+{\left(-1\right)}^{m}S_{m}+{\left(-1\right)}^{d_{\alpha }}S_{add}^{\left(d_{\alpha }-d_{\beta }\right)}\right)\right)dt$$

However, the variation can be identically equal to zero only for *d_α_* = *d_β_*, i.e., for a zero distance of the element from the Σ-diagonal. Due to its time-domain differentiation or integration, the state function *S_add_* loses the ability to be a scalar potential function, which can generate the monogenic quantity *f_add_* [1]. The generalized voltages *f_add_* ^(*d*)^ and *f_add_* ^(-*d*)^, by which the terms below and above the Σ-diagonal contribute by (32) to KV^(*α*)^L, are polygenic quantities [1] that cannot be included in the Lagrangian. That is why Hamilton’s principle holds only for *d* = 0, i.e., when all the elements of the circuit are located on the same Σ-diagonal.

The proof holds for an arbitrary position of the hidden elements on the Σ-diagonal and for an arbitrary number of elements. These parameters can be modified in order to achieve that the point, lying off the diagonal, will be situated within the characteristic quarter-circle, as illustrated in Figure 7.

## 5. Compatibility with the Classical Variational Principle

Basically, the Lagrangian (25) of the variational principle (24) can be of two different forms according to whether the circuit elements are located on the odd or the even Σ-diagonal of the table (see Figure 3).

The *L* and *C* elements occupy the odd Σ-diagonals, and the state functions *S*_0_ to *S_m_* from (25) are the potential functions (13a). Their definition formulae together with (25) provide the resulting form of the Lagrangian for a circuit built only from elements of the odd Σ-diagonal

(36) $$L=T^{*}-V$$

where T* and *V* are the potential cofunction and function, representing parts of the circuit built exclusively from the elements of *L* and *C* types. The Lagrangian can, therefore, be obtained by subtracting the sum of the generalized energies of all capacitors from the sum of the generalized coenergies of all inductors in the circuit.

The even Σ-diagonals contain the elements of ^+^*R* and ^−^*R* types, and the state functions *S*_0_ to *S_m_* from (25) are the potential functions (13b), ^+^*D* for positive, and ^−^D^*^ for negative resistive elements. Their definition formulae together with (25) give the resulting Lagrangian for a circuit built only from the elements from even Σ-diagonal

(37) L=D+−D*−

where ^+^*D* and ^−^D* are the potential function and cofunction, representing parts of the circuit built exclusively from the elements of ^+^*R* and ^−^*R* types. The Lagrangian can, therefore, be obtained by subtracting the sum of generalized dissipative cofunctions of all negative resistors from the sum of generalized dissipative functions of all positive resistors in the circuit.

Formulaes (36) and (37), defining the Lagrangian, can be modified, considering that the Euler–Lagrange equations generated by the Lagrangian do not depend on its sign. The sign of the Lagrangian only exerts influence on the type of the extreme of the action potential, which is reached during the movement along the existing trajectory.

Equations (36) and (37) hold for the current representation of the circuit. The voltage representation works with dual quantities, which means that the functions are replaced by cofunctions and vice versa [27].

## 6. Application: Pais–Uhlenbeck Oscillator

The necessary and sufficient condition for the validity of Hamilton’s principle, namely that all the circuit elements must occupy one common Σ-diagonal, strictly holds only for nonlinear elements, i.e., elements with nonlinear constitutive relations. It is well known that the character of the linear element is not changed during the element movement along the Δ-diagonal [8,32]. Such a movement changes the *α* and *β* indices by the same value *k*, thus both the original (*F*(*v*^(*α*)^,*i*^(*β*)^) = 0) and the new (*F*(*v*^(*α*+*k*)^,*i*^(*β*+*k*)^) = 0) relations hold simultaneously. The linear (*α*,*β*) element can therefore be considered as an arbitrary (*α* + *k*,*β* + *k*) element for an arbitrary integer *k*. Taking also into account the linear elements, one has a chance to escape from the strict rule of one Σ-diagonal. The procedure is illustrated on the example of the synthesis of the Pais–Uhlenbeck (PU) oscillator via higher-order elements. The PU oscillator [33] is a toy model of recent higher-derivative theories. Its Lagrangian and differential equation are adopted from [34].

The PU oscillator is described by the second-order Lagrangian

(38) $$L\left(x,\dot{x},\ddot{x}\right)=-\frac{\epsilon m}{2\omega^{2}}{\ddot{x}}^{2}+\frac{m}{2}{\dot{x}}^{2}-\frac{m\omega^{2}}{2}x^{2}$$

and the resulting trajectory *x*(*t*) is the extremal of the action functional, which is based on the Lagrangian (38). The Euler–Lagrange equation, corresponding to this Lagrangian, is

(39) $$\frac{\epsilon }{\omega^{2}}\ddddot{x}+\ddot{x}+\omega^{2}x=0$$

Equation (39) is a linear differential equation with constant parameters with the solution

(40) $$x\left(t\right)=C^{+}\cos\left(\omega k^{+}t\right)+S^{+}\sin\left(\omega k^{+}t\right)+C^{-}\cos\left(\omega k^{-}t\right)+S^{-}\sin\left(\omega k^{-}t\right)$$

where

(41) $$k^{\pm }=\sqrt{\frac{1\mp \sqrt{1-4\epsilon }}{2\epsilon }}$$

and *C*^+^, *C*^–^, *S*^+^, and *S*^–^ are constants given by the initial conditions. The system, therefore, oscillates at two different frequencies. For ϵ = 0, it changes to a classical harmonic oscillator with the oscillation frequency *ω*.

Since the oscillator fulfills Hamilton’s principle, it should be synthetized via higher-order elements. Comparing the differential equation (39) and the relation (26), the synthesis can be performed via three elements with the constitutive relations

(42) $$f_{0}\left(x\right)=\omega^{2}x,f_{1}\left(\dot{x}\right)=\dot{x},f_{2}\left(\ddot{x}\right)=\frac{\epsilon }{\omega^{2}}\ddot{x}$$

**Figure 8:**
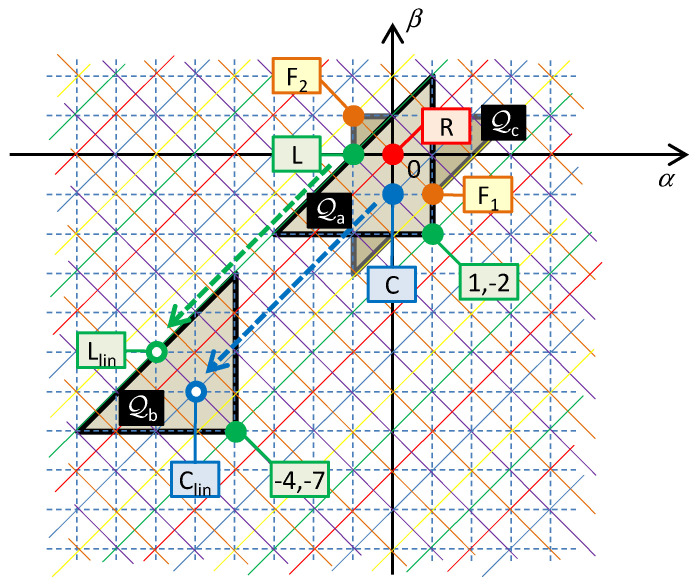
Quarter-circles for three elements used for the synthesis of various versions of the Pais–Uhlenbeck oscillator. *Q*_a_—series connection of the *R*, *L*, and (1,−2) elements, *Q*_b_—series connection of the hypothetical (−4,−7) element and linear *R* and *L*, *Q*_c_—parallel connection of resistor and two different types of FDNR; *F*_1_/*F*_2_—negative resistance decreasing/increasing with the square of frequency. The oscillating quantity *x* from (39) for the series/parallel connection is the current/voltage coordinate with the order given by the position of the hidden element in the table.

All three elements will neighbor on an arbitrary common Σ-diagonal. The generalized coordinate *x* (current variable for the series connection) and the physical size of generalized forces (voltage variables of individual in-loop elements) will be given by the specific positioning of the hidden element in Chua’s table (see Figure 8).

**Figure 9:**
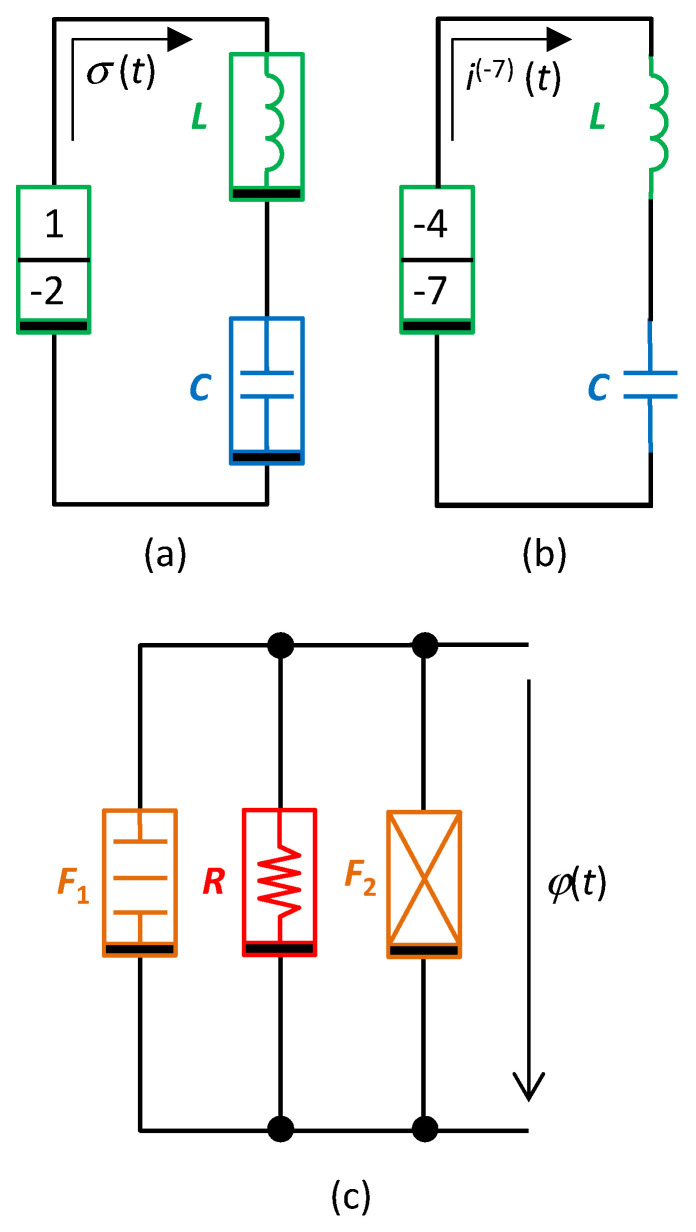
Examples of various implementations of the Pais–Uhlenbeck oscillator via HOEs. The variable *x* from Equation (39) or (43) is *σ*(*t*), *i*^(−7)^(*t*), or *ϕ*(*t*). (a) Nonlinear elements allow synthetizing the oscillator with the differential equation (43), (b) implementation of the oscillator via the linear *L* and *C* elements, (c) generally nonlinear oscillator (43) with voltage output; *F*_1_ = FDNC, *F*_2_ = FDNR, schematic symbols are in accordance with [12].

Figure 8 illustrates the characteristic quarter-circles *Q*_a_, *Q*_b_, and *Q*_c_, which represent three different implementations of the oscillator in Figure 9a–c. The given characteristic quarter-circles for series and parallel connections confirm that the order of the circuits is always four [28].

*Q*_a_ is the quarter-circle for the series connection of the inductor *L*, capacitor *C*, and the (1,−2) element, whose small-signal model represents the inductance decreasing with the fourth power of frequency. In this case, the variable *x* is the double time-domain integral of current or the integral of charge *σ* = ∫*qdt*. For nonlinear elements, i.e., elements whose constitutive relations *f*_0_(), *f*_1_(), and *f*_2_() are nonlinear functions, the series circuit in Figure 9a models the nonlinear PU oscillator, governed by the nonlinear differential equation

(43) $${\ddot{f}}_{2}\left(\ddot{x}\right)+{\dot{f}}_{1}\left(\dot{x}\right)+f_{0}\left(x\right)=0$$

The Lagrangian is a nonlinear form of the Lagrangian (37) as a sum of the coenergies of the inductive elements *L* and the (1,−2) minus the energy of the capacitor:

(44) $$L\left(x,\dot{x},\ddot{x}\right)=\int f_{0}\left(x\right)dx-\int f_{1}\left(\dot{x}\right)d\dot{x}+\int f_{2}\left(\ddot{x}\right)d\ddot{x}$$

*Q*_b_ in Figure 8 is the quarter-circle for the series connection of the (−4,−7) element, which is concurrently the hidden element, and two other nearest neighbors of *C* and *L* types on the corresponding Σ-diagonal. If these two elements are linear, their character does not change during their movement along the Δ-diagonal. That is why the linear capacitor *C* and the linear inductor *L* can be used according to Figure 9b. In this case, the quantity *x* is the sevenfold time-domain integral of current or the quantity *i*^(−7)^(*t*). For a nonlinear (−4,−7) element, the Lagrangian is in the form

(45) $$L\left(x,\dot{x},\ddot{x}\right)=\int f_{0}\left(x\right)dx-\frac{m}{2}{\dot{x}}^{2}+\frac{\epsilon m}{2\omega^{2}}{\ddot{x}}^{2}$$

where the right-side terms of (45) are the coenergy of the (−4,−7) element, energy of the capacitor, and coenergy of the inductor, respectively.

If the differential equation (43) represents the equilibrium of the generalized velocities and not the equilibrium of generalized forces as we hitherto supposed, it can be considered the generalized Kirchhoff law for the current variables *i*^(*β*)^, and this equation can be modeled by a parallel connection of (*α*,*β*) elements.

Figure 8 shows the quarter-circle *Q*_c_ for a parallel connection of the resistor *R* and two elements, *F*_1_ and *F*_2_, namely the frequency-dependent negative resistors. *F*_1_ is the classical FDNC [12], whose small-signal resistance decreases with the square of the frequency. *F*_2_ is the FDNR with opposite dependence, i.e., its resistance increases with the square of the frequency [12]. Comparing these effects with the frequency-dependent terms of the differential equation (39) yields the explanation of why the given circuitry can generate the solution of the equation of just this type. Removing the *F*_2_ element, which represents the first term of the differential equation (39) or (43), leads to the classical harmonic oscillator for ϵ = 0.

If all three elements are nonlinear, then the Lagrangian has a general form (44). The individual right-side terms of (44) mean the content of the FDNR, cocontent of the resistor, and content of the FDNC.

## 7. Discussion

It is proved that Hamilton’s variational principle holds for circuits built from higher-order elements that occupy the common Σ-diagonal of Chua’s table. Furthermore, it is shown that this principle is voided if any other element outside this diagonal appears in the circuit. For the case of elements located on the odd Σ-diagonal, the Lagrangian is the difference between the generalized energies of the elements of *L* and *C* types. For the even Σ-diagonal, it is the difference between the generalized contents of the elements of ^+^*R* and ^−^*R* types. Created in this way, the Lagrangian has the form (7), and the equations of motion are always of the even order. This follows from the way of drawing the Lagrangian via integrals of the constitutive relations of individual elements. If the linear elements are present in the circuit, each contributes to equations of motion only by the even order of the derivative of the generalized variable.

An arbitrary quantity, used for the circuit description in the frame of a given method of circuit analysis, can be chosen as a generalized variable in the Lagrangian. This fact is demonstrated by means of several examples of the synthesis of the Pais–Uhlenbeck oscillator via higher-order elements that are located on a common Σ-diagonal. To preserve the strict notation, it is necessary to allow for a consequence of voltage-current duality, namely that cofunctions and co-Lagrangians correspond to potential functions and Lagrangians [27].

## Figures and Tables

**Table 1:** Classifying the state functions of current and voltage representations (12) into functions and cofunctions. The cofunctions are denoted by asterisks.

Type of the Element	^+^ *R*	*L*	^−^ *R*	*C*
Current representation *S_α_*_,_*_β_*	^+^ *D_α_*_,_ *_β_*	*T* ^*^ *_α_*_,_ *_β_*	^−^ *D* ^*^ *_α_*_,_ *_β_*	*V_α_*_,_ *_β_*
Voltage representation *Ŝ_α_*_,_*_β_*	^+^ *D* ^*^ *_α_*_,_ *_β_*	*T_α_*_,_ *_β_*	^−^ *D_α_*_,_ *_β_*	*V* ^*^ *_α_*_,_ *_β_*

**Table 2:** Uniform notation for circuit description via both Kirchhoff’s Laws.

Law	Hidden Element	*x*	*u*	DE
KV^(*α*)^L	$\left(\alpha_{\mathrm{MAX}},\beta_{\mathrm{MIN}}\right)$	$i^{\left(\beta_{\mathrm{MIN}}\right)}$	$v^{\left(\alpha_{\mathrm{MAX}}\right)}$	$\sum_{j=0}^{m}f_{j}^{\left(\alpha_{\mathrm{MAX}}-\alpha_{j}\right)}\left(x^{\left(\beta_{j}-\beta_{\mathrm{MIN}}\right)}\right)=u$
KC^(*β*)^L	$\left(\alpha_{\mathrm{MIN}},\beta_{\mathrm{MAX}}\right)$	$v^{\left(\alpha_{\mathrm{MIN}}\right)}$	$i^{\left(\beta_{\mathrm{MAX}}\right)}$	$\sum_{j=0}^{m}g_{j}^{\left(\beta_{\mathrm{MAX}}-\beta_{j}\right)}\left(x^{\left(\alpha_{j}-\alpha_{\mathrm{MIN}}\right)}\right)=u$
